# Comparative analysis of haplotypes carrying pathogenic variants c.1545T>G, c.2027T>A and c.919-2A>G of the SLC26A4 gene in patients with hearing loss from the Tyva Republic (Southern Siberia)

**DOI:** 10.18699/vjgb-25-17

**Published:** 2025-02

**Authors:** V.Yu. Danilchenko, M.V. Zytsar, E.A. Panina, K.E. Orishchenko, O.L. Posukh

**Affiliations:** Institute of Cytology and Genetics of the Siberian Branch of the Russian Academy of Sciences, Novosibirsk, Russia Novosibirsk State University, Novosibirsk, Russia; Institute of Cytology and Genetics of the Siberian Branch of the Russian Academy of Sciences, Novosibirsk, Russia Novosibirsk State University, Novosibirsk, Russia; Institute of Cytology and Genetics of the Siberian Branch of the Russian Academy of Sciences, Novosibirsk, Russia Novosibirsk State University, Novosibirsk, Russia; Institute of Cytology and Genetics of the Siberian Branch of the Russian Academy of Sciences, Novosibirsk, Russia Novosibirsk State University, Novosibirsk, Russia; Institute of Cytology and Genetics of the Siberian Branch of the Russian Academy of Sciences, Novosibirsk, Russia Novosibirsk State University, Novosibirsk, Russia

**Keywords:** hearing loss, SLC26A4, pathogenic variants, STRs, SNPs, haplotypes, founder effect, Siberian populations, потеря слуха, SLC26A4, патогенные варианты, STR, SNP, гаплотипы, эффект основателя, популяции Сибири

## Abstract

Pathogenic variants in the SLC26A4 gene (OMIM #605646), leading to non-syndromic recessive hearing loss type 4 (DFNB4) and Pendred syndrome, significantly contribute to the etiology of hearing loss in many populations of the world. The spectrum and prevalence of different pathogenic SLC26A4 variants are characterized by wide ethnogeographical variability. A high frequency of some of them in certain regions of the world may indicate either their independent origin or be a consequence of the founder effect. The proportion of SLC26A4-associated hearing loss in Tuvinian patients (the Tyva Republic, Southern Siberia) is one of the highest in the world (28.2 %) and the vast majority of mutant SLC26A4 alleles are represented by three pathogenic variants c.919-2A>G, c.2027T>A and c.1545T>G (69.3, 17.5 and 8.0 %, respectively). Their overall carrier frequency in the Tuvinian population reaches 7.1 %. The accumulation of these variants in Tuvinian patients suggests a role of the founder effect in their prevalence in Tuva, which can be confirmed by the common genetic background (haplotypes) for each of them. For reconstruction of haplotypes in the carriers of variants c.1545T>G and c.2027T>A, the genotyping data of a panel of polymorphic genetic markers were used: five STRs (four of them flank the SLC26A4 gene at different distances and one is intragenic) and nine intragenic SNPs. Comparative analysis of the reconstructed haplotypes for c.1545T>G and c.2027T>A with previously obtained data on haplotypes for the c.919-2A>G variant showed that each of the analyzed variants has a specific (similar for all carriers of a particular variant) genetic background, apparently inherited from different “founder ancestors”. These data confirm the cumulative founder effect in the prevalence of pathogenic variants c.1545T>G, c.2027T>A, and c.919- 2A>G of the SLC26A4 gene in the indigenous population of the Tyva Republic. The obtained data are relevant both for predicting the prevalence of SLC26A4-caused hearing loss and for development of region-specific DNA diagnostics of inherited hearing loss in the Tyva Republic.

## Abstract (ru)

Патогенные варианты в гене SLC26A4 (OMIM #605646), приводящие к несиндромальной рецессивно наследуемой потере слуха 4-го типа (DFNB4) и синдрому Пендреда, вносят весомый вклад в этиологию потери слуха во многих популяциях мира. Спектр и распространенность различных патогенных вариантов гена SLC26A4 характеризуются широкой этногеографической вариабельностью. Высокая частота некоторых из них в отдельных регионах мира может свидетельствовать об их независимом возникновении или же быть следствием эффекта основателя. Доля SLC26A4-ассоциированной потери слуха у тувинских пациентов (Республика Тыва, Южная Сибирь) является одной из самых высоких в мире (28.2 %). Подавляющее большинство мутантных SLC26A4-аллелей представлено тремя патогенными вариантами – c.919-2A>G, c.2027T>A и c.1545T>G (69.3, 17.5 и 8.0 % соответственно). Суммарная частота их гетерозиготного носительства в тувинской популяции достигает 7.1 %. Накопление этих вариантов у тувинских пациентов позволяет предположить роль эффекта основателя в их распространенности на территории Тувы, что может быть подтверждено общностью генетического окружения (гаплотипов) для каждого из них. Для реконструкции гаплотипов у носителей вариантов c.1545T>G и c.2027T>A были использованы данные генотипирования панели полиморфных генетических маркеров: пяти STR-маркеров (четыре из них фланкируют на разном расстоянии ген SLC26A4 и один является внутригенным) и девяти внутригенных SNP-маркеров. Сравнительный анализ реконструированных гаплотипов для c.1545T>G и c.2027T>A с ранее полученными данными о гаплотипах для c.919-2A>G показал, что каждый из анализируемых вариантов имеет особое и сходное для всех носителей того или иного варианта генетическое окружение, по-видимому, унаследованное от различных «предков-основателей». Эти данные подтверждают роль кумулятивного эффек- та основателя в распространенности патогенных вариантов с.1545T>G, c.2027T>A и c.919-2A>G гена SLC26A4 у коренного населения Республики Тыва. Полученные данные актуальны как для прогнозирования распространенности SLC26A4-обусловленной потери слуха, так и для создания регион-специфичной ДНК-диагностики наследуемой потери слуха в Республике Тыва.

## Introduction

Currently, more than 5 % of the world’s population has severe or profound hearing loss, caused by both environmental and genetic factors (World Health Organization, https:// www.who.int/ru). Genetic factors underlie more than half of all cases of congenital (or early manifestation) pathology of auditory function. Hereditary hearing loss can be one of the clinical features of many (about 400) syndromes, or an isolated (non-syndromic) pathology, which is characterized by unique genetic heterogeneity: about 200 loci have already been mapped and at least 150 genes associated with hearing loss have been identified (Hereditary Hearing Loss Homepage: https://hereditaryhearingloss.org, April 2024).

Wide ethnogeographical variability is known in the prevalence of various forms of inherited hearing loss caused by pathogenic variants in different “deafness genes”. The “accumulation” of some forms of hereditary hearing loss in a certain population, like a number of other monogenic diseases, can be determined by the ethnic composition of the population, isolation, marital structure, founder and bottleneck effects, as well as a possible selective advantage of heterozygotes (Scott et al., 1995; Ben Arab et al., 2004; Common et al., 2004; Zlotogora, 2007; Chong et al., 2012; Razdan et al., 2012). An important role in the prevalence of hereditary forms of deafness was probably also played by such a social factor as the long-term tradition of assortative marriages between deaf people, based on their linguistic homogamy (sign language), which led to an increase in social adaptation and genetic fitness of deaf people (Nance et al., 2000; Nance, Kearsey, 2004).

Identification of the most frequent (major) mutations in genes involved in hearing loss is an urgent task for both genetic risk assessment and medical and genetic counseling of affected families, and for developing the most effective methods of molecular diagnostics of this pathology. The high frequency of some mutations in certain regions of the world may either indicate their independent occurrence (mutational “hot spot”) or be a consequence of the founder effect (founder mutation). The founder effect in the prevalence of mutations can be confirmed by the similarity of their genetic background (haplotypes). Haplotype reconstruction is usually carried out based on the analysis of highly polymorphic genetic markers: STRs (Short Tandem Repeats) and SNPs (Single Nucleotide Polymorphisms). Analysis of haplotypes carrying a particular mutation can allow to estimate its “age” (time of occurrence) using a “molecular clock” approach, and, in some cases, identify potential regions of its origin using information about population history.

The most significant contribution to the etiology of hearing loss in many populations of the world is made by pathogenic variants in the GJB2 gene (OMIM #121011). The SLC26A4 gene (solute carrier family 26, member 4, 7q22.3, OMIM #605646) is the second most significant gene, at least for Asian populations. SLC26A4 encodes the transmembrane transport protein pendrin, which is involved in the transport of various ions and is mainly expressed in the inner ear, thyroid gland and kidneys. Pathogenic variants in the SLC26A4 gene lead to non-syndromic recessive hearing loss (type DFNB4) and Pendred Syndrome (OMIM #274600), a recessive disease characterized by hearing loss and goiter. Patients with SLC26A4-associated hearing loss often have abnormalities of the bony labyrinth of the inner ear (enlarged vestibular aqueduct, Mondini dysplasia). Numerous studies have found that the prevalence of SLC26A4-associated hearing loss and the spectrum of pathogenic variants of this gene vary significantly in different regions of the world. It has now become apparent that the spectrum of pathogenic variants of the SLC26A4 gene found in Asian populations is significantly different from that in populations of European origin (Park et al., 2003; Albert et al., 2006; Du et al., 2013; Lu Y.J. et al., 2015; Tsukada et al., 2015).

Analysis of the SLC26A4 gene, conducted during long-term studies of inherited hearing loss in Tuvinians, the indigenous population of the Tyva Republic (Southern Siberia), showed that the proportion of SLC26A4-associated hearing loss in Tuvinian patients is one of the highest in the world (28.2 %) (Danilchenko et al., 2021). A specific spectrum of variations in the SLC26A4 gene was identified in Tuvinian patients, including both known pathogenic variants and a number of new variants with still uncertain clinical significance. The vast majority of mutant SLC26A4 alleles identified in patients were represented by three pathogenic variants c.919-2A>G, c.2027T>A and c.1545T>G (69.3 %, 17.5 %, and 8.0 %, respectively), and their overall carrier frequency reached 7.1 % in the Tuvinian control sample (Danilchenko et al., 2021). The predominance of variant c.919-2A>G suggested the role of the founder effect in its accumulation in Tuvinians, and in our recent study (Danilchenko et al., 2023), we identified a similarity of STR and SNP haplotypes in all c.919-2A>G carriers, which convincingly indicates its origin from a common ancestor, thereby confirming the decisive role of the founder effect in the prevalence of this pathogenic SLC26A4 variant in the indigenous population of the Tyva Republic

The aim of this study is a comparative analysis of the genetic background of pathogenic variants c.1545T>G, c.2027T>A, and c.919-2A>G of the SLC26A4 gene, identified with high frequency in the indigenous population of the Tyva Republic.

## Materials and methods

Analyzed samples. Genotyping of genetic markers (STRs and SNPs) for the analysis of haplotypes of the chromosome 7 region including the SLC26A4 gene was performed on the sample of Tuvinian patients having variant c.2027T>A in a homozygous (n = 4) or compound heterozygous (n = 15) state, or variant c.1545T>G in a compound heterozygous state (n = 15). For comparative analysis, previously obtained data on the structure of haplotypes in Tuvinian patients homozygous for the c.919- 2A>G variant of the SLC26A4 gene (n = 23) and in individuals from the control sample represented by unrelated Tuvinians (n = 63) were used (Danilchenko et al., 2023). Written informed consent was obtained from all individuals or their legal guardians before venous blood sampling for DNA extraction. The study was approved by the Bioethics Commission at the Institute of Cytology and Genetics of the Siberian Branch of the Russian Academy of Sciences (Novosibirsk, Russia).

Historical information about the Tuvinian population. Tuvinians (Tuvans), numbering about 300,000 people in total (according to the 2021 All-Russian Census), currently live mainly in the Tyva Republic, which borders Mongolia to the south and east. In addition to the Tyva Republic and several other regions of Russia, relatively small groups of Tuvinians also live in northern Mongolia and in Xinjiang Uyghur Autonomous Region of China (Mongush, 1996; Chen et al., 2011).

Tuvinians are one of the oldest Turkic-speaking peoples inhabiting Central Asia and the Sayan-Altai region. At different times, Tuva was at the periphery of a powerful state of Huns (2nd century BC – 1st century AD), was part of the Ancient Turkic Khaganate (6–8th centuries), of the Uyghur Khaganate (8–9th centuries), of the Yenisei Kyrgyz Khaganate (9–12th centuries), and was also incorporated in the Mongol Empire (13–14th centuries). These historical events, as well as long-term contacts with the population of neighboring regions (Turkic-, Mongolic-, Ket-, and Samoyedic-speaking tribes), had a certain influence on the formation of the Tuvinian ethnic group (Vainshtein, Mannay-Ool, 2001; Mannai-ool, 2004) and the genetic structure of the Tuvinian population.

**Fig. 1.**
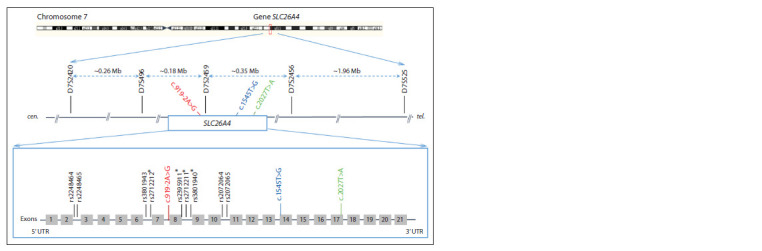
Schematic structure of the SLC26A4 gene and the location of genetic markers (five STRs and nine SNPs) which were used to reconstruct the haplotypes Pathogenic variants c.919-2A>G, c.1545T>G and c.2027T>A of the SLC26A4 gene are highlighted in color. The distances between STRs (bp) are given in megabases (Mb). * – four SNPs from (Wu et al., 2005) used for comparative analysis. cen. – centromere, tel. – telomere. The figure was modified from (Danilchenko et al., 2023).

Experimental methods. To analyze the haplotypes carrying variants c.1545T>G and c.2027T>A of the SLC26A4 gene, we performed the genotyping of genetic markers (STRs and SNPs), which we had previously used in our study to investigate the structure of haplotypes for variant c.919-2A>G (Danilchenko et al., 2023): five STRs (D7S2420, D7S496, D7S2456, D7S525, flanking the SLC26A4 gene at different distances, and intragenic D7S2459), as well as nine intragenic SNPs (rs2248464, rs2248465, rs3801943, rs2712212, rs2395911, rs2712211, rs3801940, rs2072064, rs2072065) (Fig. 1).

Genotyping of STRs (fragment analysis) and SNPs (Sanger sequencing) was performed on an ABI 3130XL genetic analyzer (Applied Biosystems, USA) in the SB RAS Genomics Core Facility (Institute of Chemical Biology and Fundamental Medicine SB RAS, Novosibirsk, Russia). Details of the experimental genotyping methods are presented in the work (Danilchenko et al., 2023).

Statistical methods. A one-sided Fisher’s exact test with a significance level of p < 0.05 was applied to compare the allele and haplotype frequencies between the examined samples

Linkage disequilibrium between the alleles of STR markers of chromosome 7 and alleles with variants c.1545T>G or c.2027T>A of the SLC26A4 gene was calculated using the formula δ = (Pd – Pn) / (1 – Pn), where δ is a measure of linkage disequilibrium; Pd is the frequency of the associated allele among chromosomes with variants c.1545T>G or c.2027T>A in the samples of patients; Pn is the frequency of the same allele among chromosomes without these variants in the control sample (Bengtsson, Thomson, 1981).

Reconstruction of haplotypes based on the detected alleles of STR and SNP markers in samples of the carriers of variants c.1545T>G or c.2027T>A was carried out manually, using data from the analysis of genetic markers in their relatives (when it was possible). Reconstruction of haplotypes and analysis of their frequency distribution in the control sample of Tuvinans were performed by us using software package Arlequin v.3.5.1.2 (https://cmpg.unibe.ch/software/arlequin3512/, Expectation-Maximization algorithm) (Danilchenko et al., 2023).

Estimation of the “age” of variants of the SLC26A4 gene. Estimation of the “age” of a mutation is based on the expected loss of linkage between the mutation and alleles of surrounding genetic markers over time due to recombination (the “molecular clock” concept). To estimate the “age” of the analyzed variants, two methods were used: the “single marker method” based on the allelic variation of one marker (Risch et al., 1995; Slatkin, Rannala, 2000), and the second method based on haplotype data implemented by the DMLE+ v.2.3 program (Disequilibrium Mapping and Likelihood Estimation, DMLE+ v.2.3: http://dmle.org/) (details of the methods used are given in Supplementary Material 1)1. The “age” of a variant was determined by estimating the number of generations (g) and years (assuming that g = 25 years) that have passed since its occurrence


Supplementary Materials are available in the online version of the paper: https://vavilov.elpub.ru/jour/manager/files/Suppl_Danil_Engl_29_1.pdf


## Results

In our recent study (Danilchenko et al., 2021), we analyzed the SLC26A4 gene using Sanger sequencing in patients with hearing loss belonging to the Tuvinians, an indigenous Siberian Turkic-speaking people (the Tyva Republic, Southern Siberia). Biallelic pathogenic SLC26A4 variants were detected in 28.2 % (62 out of 220) of the patients included in the study. This rate of SLC26A4-associated hearing loss was one of the highest among all populations in the world. The vast majority of the detected mutant SLC26A4 alleles were represented by three pathogenic variants c.919-2A>G, c.2027T>A, and c.1545T>G.

Variant c.919-2A>G

Most Tuvinian patients were homozygous or compound heterozygous for pathogenic variant c.919-2A>G. The proportion of c.919-2A>G was 69.3 % among all mutant SLC26A4 alleles identified in Tuvinian patients, and its carrier frequency in the Tuvinian population was 5.1 % (Danilchenko et al., 2021). Variant c.919-2A>G is located in the canonical (-2) 3′ splice acceptor site in the intronic region between exons 7 and 8 and leads to splicing abnormalities (Yang J.J. et al., 2005; Lu Y.C. et al., 2011; Wasano et al., 2020).

Numerous studies have shown that the c.919-2A>G variant is highly prevalent in patients from Asian regions (mainland China, Taiwan, Mongolia, Korea, and Japan) and is found with the highest frequency in China and Mongolia, while in other regions of the world, this variant is extremely rare or absent (Park et al., 2003; Wu et al., 2005; Albert et al., 2006; Dai et al., 2008; Du et al., 2013; Yang X.L. et al., 2013; Lu Y.J. et al., 2015; Tsukada et al., 2015; Erdenechuluun et al., 2018).

Variant c.2027T>A

Variant c.2027T>A (p.Leu676Gln) of the SLC26A4 gene was found in homozygous or compound heterozygous state in 19 Tuvinian patients and was the second most frequent pathogenic variant (17.5 %) after c.919-2A>G (69.3 %) among all mutant variants of the SLC26A4 gene identified in Tuvinian patients (Danilchenko et al., 2021).

Variant c.2027T>A leads to the replacement of leucine with glutamine at amino acid position 676 (p.Leu676Gln) in the highly conserved region of the STAS domain in the COOHterminal part of the pendrin protein molecule. Experimental studies have shown that this variant leads to retention of the mutant protein in the intracellular space and disruption of its function (Gillam et al., 2004; Yoon et al., 2008). The c.2027T>A variant was detected at low frequency (only in isolated patients in compound heterozygous or heterozygous state) in China, Korea, and Mongolia (Park et al., 2003; Choi et al., 2009; Huang et al., 2011; Chai et al., 2013; Erdenechuluun et al., 2018).

Variant c.1545T>G

Variant c.1545T>G is a new, previously undescribed missense variant in exon 14 of the SLC26A4 gene, apparently resulting in the substitution of phenylalanine with leucine at amino acid position 515 (p.Phe515Leu), was found in a compound heterozygous state in 15 Tuvinian patients originating from ten unrelated families. The carrier frequency of c.1545T>G in the Tuvinian control sample was 2.0 %. Segregation of c.1545T>G with hearing loss in the pedigrees of patients, a significant excess of its frequency in the sample of patients compared to the control sample ( p = 0.03391), the results of predictive computer programs and the absence of this variant in the world’s human genomic databases support its pathogenic significance (Danilchenko et al., 2021).

Reconstruction of STR haplotypes for variants c.1545T>G and c.2027T>A of the SLC26A4 gene

To reconstruct the haplotypes of the chromosome 7 region carrying pathogenic variants c.1545T>G and c.2027T>A of the SLC26A4 gene, five STRs (D7S2420, D7S496, D7S2459, D7S2456, D7S525) were genotyped in unrelated carriers of these variants (Fig. 1). These STRs were previously used in the analysis of haplotypes carrying the pathogenic variant c.919- 2A>G (Danilchenko et al., 2023). The results of genotyping of STR markers, in comparison with the data obtained on the Tuvinian control sample, are presented in Supplementary Material 2 (Tables S1 and S2). All STRs in the control sample were previously found to be highly polymorphic: D7S2420 – 10 alleles, D7S496 – 10 alleles, D7S2459 – 7 alleles, D7S2456 – 5 alleles, D7S525 – 8 alleles (Danilchenko et al., 2023). All STRs in the carriers of variant c.1545T>G were monomorphic (only one allele for each STR marker) (Table S1). In the carriers of variant c.2027T>A, four STRs were monomorphic (D7S2420, D7S496, D7S2459, and D7S2456), but for the distal marker D7S525, three different alleles (221, 227, 231) were detected, with frequencies of 0.4000, 0.1333 and 0.4667, respectively (Table S2). Comparative analysis of the frequencies of D7S525 alleles in the carriers of c.2027T>A and in the control sample revealed statistically significant differences ( p < 0.05) in the frequencies of alleles 227 and 231 (Table S2).

Genotyping of STRs in the carriers of variant c.1545T>G revealed complete linkage of this variant with alleles 286 (D7S2420), 118 (D7S496), 147 (D7S2459), 244 (D7S2456), 229 (D7S525); thus, variant c.1545T>G is characterized by a single haplotype 286-118-147-с.1545T>G-244-229, the size of which, determined by distal markers D7S2420 and D7S525, is ~ 2.75 Mb. STRs analysis in the carriers of c.2027T>A revealed complete linkage of this variant with alleles 280 (D7S2420), 118 (D7S496), 141 (D7S2459), 244 (D7S2456), but the presence of three different alleles (221, 227, 231) at the distal marker D7S525 suggests the presence of three different haplotypes for variant c.2027T>A.

**Fig. 2.**
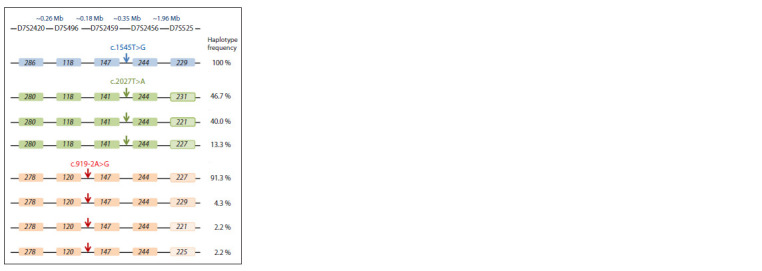
Schematic representation of the STR haplotypes in the carriers of variants c.1545T>G and c.2027T>A in comparison with the STR haplotypes for variant c.919-2A>G (Danilchenko et al., 2023). The localization of each of the analyzed variants (c.1545T>G, c.2027T>A or c.919-2A>G) is shown by an arrow.

STR haplotypes reconstructed for variants c.1545T>G and c.2027T>A, in comparison with STR haplotypes for variant c.919-2A>G, are presented in Figure 2.

We compared the structure and frequency of the STR haplotypes found for variants c.1545T>G and c.2027T>A with the STR haplotypes that were previously identified for variant c.919-2A>G (Danilchenko et al., 2023) (Table 1). It should be noted that the STR haplotypes found for all three variants c.1545T>G, c.2027T>A and c.919-2A>G differ in allelic composition, which indicates a pronounced specificity of the genetic background for each of them. In addition, a comparison of the frequency of the main STR haplotypes in the samples of carriers of c.1545T>G, c.2027T>A and c.919- 2A>G (groups of Tuvinian patients with hearing loss) and in the Tuvinian control sample revealed statistically significant differences (Table 1).

**Table 1.**
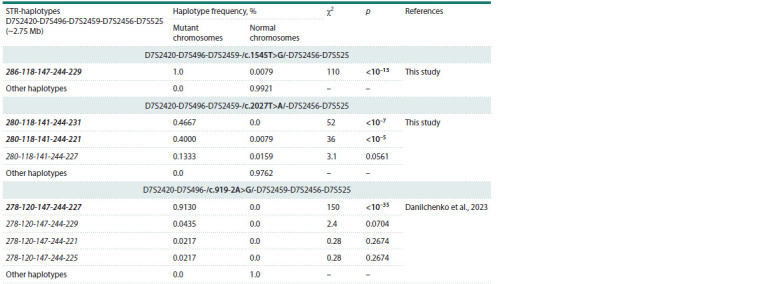
The frequencies of STR haplotypes found on the mutant chromosomes carrying pathogenic variants c.919-2A>G, c.1545T>G or c.2027T>A of the SLC26A4 gene, compared with the normal chromosomes Notе. Designations of the STR alleles included in haplotypes correspond to the size of the PCR products (in nucleotides). The most common haplotypes and statistically significant (p < 0.05) differences in haplotype frequencies are shown in bold.

Reconstruction of SNP haplotypes for variants c.1545T>G and c.2027T>A of the SLC26A4 gene

**Fig. 3.**
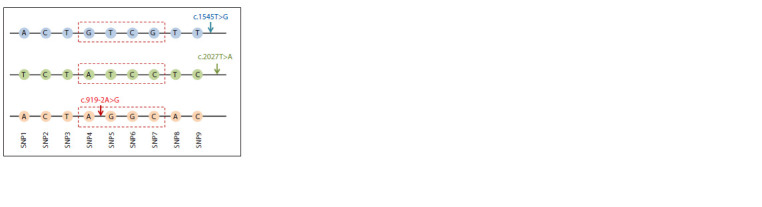
Schematic representation of SNP haplotypes in the carriers of variants c.1545T>G and c.2027T>A in comparison with SNP haplotypes for variant c.919-2A>G (Danilchenko et al., 2023). SNP marker designations: SNP1 – rs2248464, SNP2 – rs2248465, SNP3 – rs3801943, SNP4 – rs2712212, SNP5 – rs2395911, SNP6 – rs2712211, SNP7 – rs3801940, SNP8 – rs2072064, SNP9 – rs2072065. The red dotted lines highlight the four SNP markers analyzed in the carriers of variant c.919-2A>G in Taiwan (Wu et al., 2005). The localization of each of the analyzed variants (c.1545T>G, c.2027T>A, or c.919-2A>G) is shown by an arrow.

To study the fine structure of haplotypes including variants c.1545T>G or c.2027T>A of the SLC26A4 gene, nine intragenic SNPs (rs2248464, rs2248465, rs3801943, rs2712212, rs2395911, rs2712211, rs3801940, rs2072064, and rs2072065) were genotyped in the carriers of these variants. These SNPs were previously analyzed to reconstruct the genetic background (haplotypes) of variant c.919-2A>G in its homozygous carriers (Danilchenko et al., 2023). Four of them (rs2712212, rs2395911, rs2712211, and rs3801940) were included for comparative analysis with the data from the study by C.C. Wu et al. (2005), where they were used to establish the structure of haplotypes carrying variant c.919-2A>G in Taiwanese patients with hearing impairment (Fig. 1). The structure of SNP haplotypes for variants c.1545T>G and c.2027T>A is presented in Figure 3 in comparison with the SNP haplotype for variant c.919-2A>G (Danilchenko et al., 2023).

All carriers of variant c.1545T>G had a single SNP haplotype A-C-T-G-T-C-G-T-T (100 %), while the frequency of this haplotype in the Tuvinian control sample was 3.8 % (data not shown). All carriers of variant c.2027T>A also had a single SNP haplotype T-C-T-A-T-C-С-T-C (100 %), the frequency of which in the control sample was 1.7 % (data not shown). Previously, a single haplotype A-C-T-A-G-GC- A-C (100 %) was also identified in all carriers of variant c.919-2A>G, and its frequency in the Tuvinian control sample was 2.8 % (Danilchenko et al., 2023). In addition, we previously established the identity of a small (~4.5 kb) “internal” SNP haplotype A-G-G-C formed by four SNPs (rs2712212, rs2395911, rs2712211, and rs3801940) in the carriers of variant c.919-2A>G – Tuvinians (Danilchenko et al., 2023) and Han Chinese from Taiwan (Wu et al., 2005), which suggests their common origin. However, this SNP haplotype was not detected in the carriers of variants c.1545T>G and c.2027T>A (Fig. 3). Thus, we can conclude that the haplotypes formed by the alleles of SNP markers for each of the three analyzed pathogenic variants of the SLC26A4 gene are highly specific

Estimation of the “age” of variants c.1545T>G and c.2027T>A of the SLC26A4 gene

In our recent study (Danilchenko et al., 2023), two methods were used to estimate the “age” of pathogenic variant c.919- 2A>G of the SLC26A4 gene: by the “single marker method”, which is based on the analysis of alleles of the most distal markers exhibiting significant linkage disequilibrium, and by using the DMLE+ v.2.3 program, where the “age” of a variant is estimated based on the reconstructed haplotypes. In this study, we applied both of these methods to estimate the “age” of variants c.1545T>G and c.2027T>A of the SLC26A4 gene (Table 2).

**Table 2.**
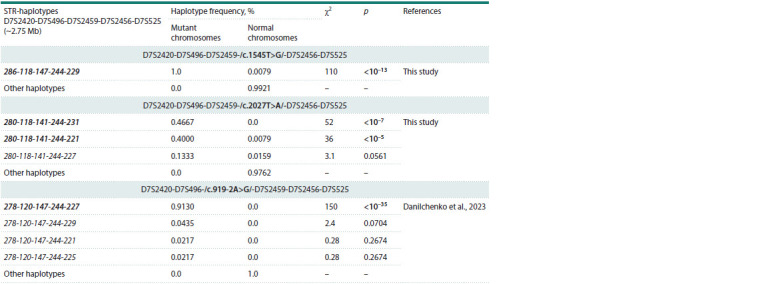
Comparative assessment of the “age” of variants c.1545T>G, c.2027T>A, and c.919-2A>G of the SLC26A4 gene based on the STR markers Notе. To assess the “age” of variants c.2027T>A and c.919-2A>G by the “single marker method”, the alleles of the distal STR marker D7S525 were used, and for the assessment by the DMLE+ v.2.3 program, STR haplotypes were used. d – different (0.05, 0.10 or 0.20) population growth rates; g – number of generations; “age” – g × 25 years; CI – confidence interval.

All carriers of variant c.1545T>G had an identical STR haplotype 286-118-147-c.1545T>G-244-229. Two haplotypes, 280-118-141-c.2027T>A-244-231 and 280-118-141-c.2027T>A-244-221, the structural differences of which are determined by the presence of different alleles (231 and 221) of the distal STR marker D7S525, were the most frequent for variant c.2027T>A (0.4667 and 0.4000, respectively). The obtained data allow us to tentatively estimate the time of occurrence of variants c.1545T>G and c.2027T>A in Tuvinians, the indigenous population of the Tyva Republic. We were unable to estimate the “age” of variant c.1545T>G using the “single marker method” due to the lack of recombination in all analyzed STR markers, but such estimates were obtained by the DMLE+ v.2.3 program (Table 2). To estimate the “age” of variant c.2027T>A by the “single marker method”, allele 231 of the distal STR marker D7S525 (~2.32 Mb from c.2027T>A), found in significant linkage disequilibrium with c.2027T>A, was used (Table S2). 

The methods used to estimate the “age” of mutations are sensitive to the demographic parameters of the population, in particular, to the population growth rates at different historical stages of its development. Since there are no accurate data on changes in the size of the indigenous population of Tuva (Tuvinians) at the early stages of its formation, we used three different population growth rates for our calculations (d = 0.05, 0.10, and 0.20) (Table 2). It should be noted that the data on the “age” of variants c.2027T>A and c.919-2A>G obtained by the “single marker method” differ from the time ranges obtained by the DMLE+ v.2.3 program, apparently “underestimating” it at all three population growth rates (d = 0.05, 0.10, 0.20). In addition, the observed overlapping of the time intervals obtained by the DMLE+ v.2.3 program for each of the analyzed variants at all population growth rates (d = 0.05, 0.10, 0.20) (Table 2) does not allow us to conclude which of the analyzed variants is “older”.

## Discussion

This work provides data on the haplotype structure for pathogenic variants c.1545T>G and c.2027T>A of the SLC26A4 gene, identified in a study of hereditary deafness in Tuvinians, the indigenous population of the Tyva Republic (Southern Siberia) (Danilchenko et al., 2021). Variant c.1545T>G was discovered for the first time; this variant has not been recorded in other regions of the world. All carriers of c.1545T>G were found to have highly specific STR and SNP haplotypes: STR haplotype 286-118-147-c.1545T>G-244-229 (100 %) and SNP haplotype A-C-T-G-T-C-G-T-T-c.1545T>G (100 %); the frequency of them in the Tuvinian control sample is less than 1 and 3.8 %, respectively. Thus, these data provide convincing evidence of a single origin of variant c.1545T>G and the role of the founder effect in its prevalence among the indigenous population of Tuva. Variant c.2027T>A is second in frequency among all pathogenic variants of the SLC26A4 gene identified in Tuvinian patients; at the same time, this variant is found only in isolated patients from China, Korea and Mongolia (Park et al., 2003; Choi et al., 2009; Chai et al., 2013; Erdenechuluun et al., 2018; Kun et al., 2024). In addition, we also found this variant in several patients from the Altai Republic, which borders the Tyva Republic (Danilchenko et al., 2021). In contrast to variant c.1545T>G, three STR haplotypes were identified in the c.2027T>A carriers: 280-118-141-c.2027T>A-244-231 (46.7 %), 280-118-141-c.2027T>A-244-221 (40.0 %), and 280-118-141-c.2027T>A-244-227 (13.3 %), that differ only by alleles of the distal STR marker D7S525.

The use of a set of polymorphic genetic markers identical to that previously used by us in the study of haplotypes of pathogenic variant c.919-2A>G of the SLC26A4 gene, the most common in Tuvinian patients (Danilchenko et al., 2023), allowed us to conduct a correct comparison of the structure of STR and SNP haplotypes for all three pathogenic variants (c.1545T>G, c.2027T>A, and c.919-2A>G). Comparative analysis showed that the composition of alleles of the genetic markers included in the haplotypes is different and highly specific for each of them. Thus, we can conclude that each of the analyzed variants has a special (similar for all carriers of a particular variant) genetic background, apparently inherited from different “founder ancestors”.

We have roughly estimated the “age” of variants c.1545T>G, c.2027T>A and c.919-2A>G, but due to the limited information on the demographic changes in the Tuvinian population throughout its history, the obtained time intervals of the appearance of these variants in the indigenous population of Tuva should be considered only as approximate ones. Nevertheless, it can be cautiously assumed that variants c.1545T>G, c.2027T>A and c.919-2A>G are not “young” (recently emerged) mutations, and the wide time intervals of their occurrence overlap at almost all population growth rates (d = 0.05, 0.10 and 0.20) (Table 2)

Data on the haplotype structure for variant c.1545T>G and its prevalence, limited only to the territory of Tuva, as well as historical information on ethnogenesis of the indigenous population of Tuva, suggest that this variant could have arisen as a result of a unique mutational event that occurred after the main formation of the Tuvinian ethnic group at the end of the 13th–14th centuries. It is more difficult to draw conclusions about the origin of variant c.2027T>A in Tuvinians. This variant is found with low frequency in patients from neighboring Mongolia and China, but, unfortunately, there are no data on the structure of the genetic background of c.2027T>A in its carriers from these regions, which excludes comparative analysis. As for variant c.919-2A>G, which is the most frequent in Tuvinians, we previously (Danilchenko et al., 2023) found the identity of the “internal” SNP haplotype A-G-G-C (Fig. 3), found in Tuvinian patients homozygous for c.919- 2A>G, and the haplotype formed by the same SNPs in the c.919-2A>G carriers from Taiwan (Han Chinese) (Wu et al., 2005). These data support the presence of a common ancestor for the “Tuvinian” and “Chinese” founder chromosomes with c.919-2A>G. Considering the obtained results, as well as the territorial distribution of variant c.919-2A>G, with a maximum frequency in Tuvinians (Southern Siberia) and in Chinese and Mongols (East and Central Asia), we suggested that variant c.919-2A>G could have arisen in geographically close territories of these regions and subsequently spread to other regions of Asia (Danilchenko et al., 2023).

## Conclusion

We analyzed the haplotype structure for pathogenic variants c.1545T>G and c.2027T>A of the SLC26A4 gene, found with high frequency in Tuvinian patients with hearing loss (the Tyva Republic, Southern Siberia). Comparative analysis of the reconstructed haplotypes for c.1545T>G and c.2027T>A with previously obtained data on the haplotypes for variant c.919- 2A>G showed that each of the analyzed variants has a specific genetic background which is similar for all carriers of a particular variant, apparently inherited from different “founder ancestors”. Thus, evidence was obtained for the role of the cumulative founder effect in the prevalence of these pathogenic variants of the SLC26A4 gene in the indigenous population of the Tyva Republic. The obtained data are relevant both for predicting the prevalence of SLC26A4-associated hearing loss and for developing region-specific DNA diagnostics of inherited hearing loss in the Tyva Republic.

## Conflict of interest

The authors declare no conflict of interest.
